# Very Severe, Not Too Severe: Reconsidering LV Dysfunction as a Barrier to Mitral TEER

**DOI:** 10.1016/j.jacadv.2026.103209

**Published:** 2026-09-23

**Authors:** Alon Shechter, David Elison

**Affiliations:** a Department of Cardiology, Rabin Medical Center, Petach Tikva, Israel; b Gray Faculty of Medical & Health Sciences, Tel Aviv University, Tel Aviv, Israel; c Division of Cardiology, Department of Medicine, University of Washington, Seattle, Washington, USA

**Keywords:** MitraClip, mitral regurgitation, mitral transcatheter edge-to-edge repair, transcatheter mitral valve repair, very severe, LV dysfunction

**Figure:**
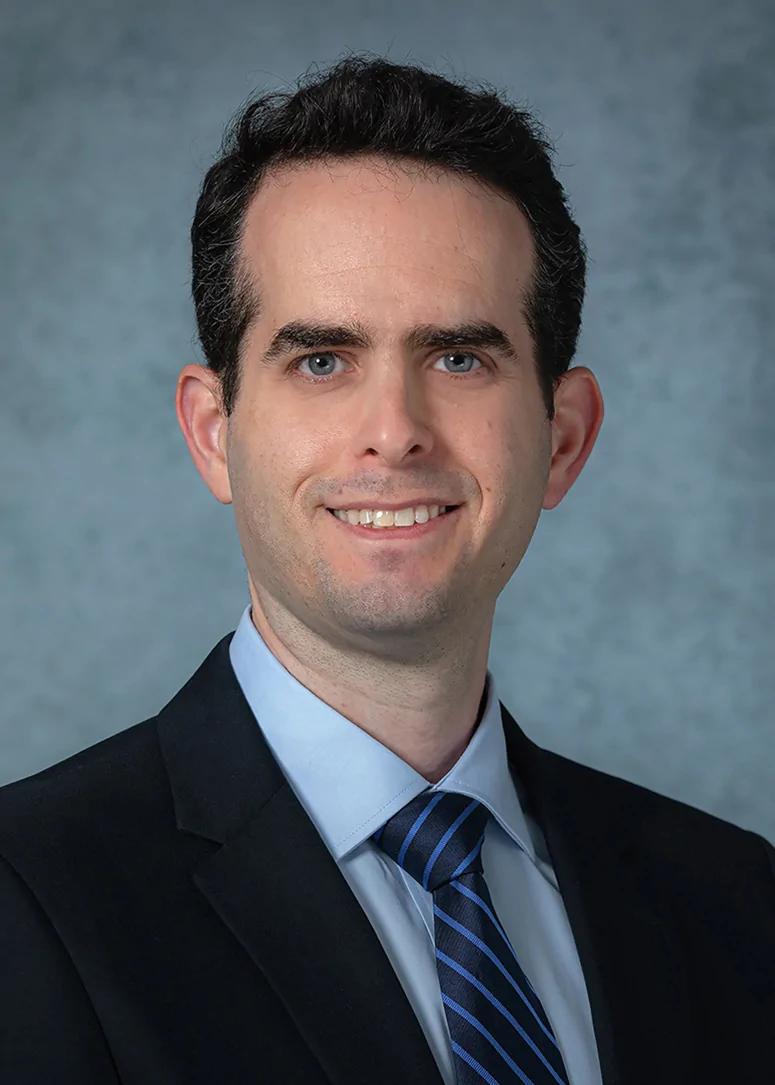

Ventricular functional mitral regurgitation (MR) (VFMR) constitutes a frequent, clinically significant consequence of left ventricular (LV) dysfunction (LVD), in which progressive remodeling leads to altered ventricular geometry, displacement of papillary muscles, and, ultimately, leaflet malcoaptation.^1^ Once established, MR further aggravates LV volume overload, accelerates remodeling, worsens symptoms, and contributes to recurrent heart failure (HF) hospitalizations and mortality. Accordingly, VFMR has emerged as a therapeutic target beyond simply as a marker of advanced HF.

Over the past decade, mitral transcatheter edge-to-edge repair (M-TEER) has transformed the management of VFMR. Following the landmark COAPT^2^ and, more recently, RESHAPE-HF2^3^ trials, the procedure has been endorsed by both American^4^ and European^5^ cardiovascular societies as an established treatment option for patients with severe disease who remain symptomatic despite maximally tolerated guideline-directed medical therapy and whose anatomical and clinical profile matches the one permitted in the randomized trials. Importantly, this leaves a growing subset of patients with very severe LVD out of the equation, as LV ejection fraction (LVEF) of <20% served as an exclusion criterion in these pivotal studies.

Aiming to fill the evidence gap surrounding M-TEER in the setting of very severe LVD were several notable observational investigations. Among the earliest, the German TRAMI registry demonstrated that severely impaired LV function (ie, an LVEF of <30%) forecasted excess 1-year mortality without compromising procedural success or symptomatic improvement.^6^ Remarkably, no distinctions were made in regard to LVD severity. Two subsequent smaller studies (n = 21 and 52) reported on improved MR burden, exercise capacity, and survival specifically in patients with very severe LVD.^7^^,^^8^ Lastly, in the largest dedicated study until recently, encompassing 96 individuals with very severe LVD (median LVEF 15%) who underwent a first-time, isolated M-TEER for chronic functional MR at Cedars-Sinai, Shechter et al^9^ observed a 95% procedural success rate and—independent of downstream LV assist device implantation and heart transplantation—significant improvement in MR grade and functional class, with NYHA functional class I-II maintained in 60% of cases by 1-year postprocedure. Furthermore, close to three-fourths of patients survived through the first postinterventional year and half did so without HF rehospitalization.

Collectively, these real-world studies suggested acceptable feasibility, safety, and efficacy of M-TEER within the very severe LVD realm. Yet an important limitation remained—as all lacked an appropriate comparator population, making it difficult to determine whether outcomes reflected the procedure itself or merely the underlying myocardial disease.

In this issue of *JACC: Advances*, Omote et al^10^ offer the much-needed missing piece of the puzzle. Using data from the Japanese OCEAN-Mitral registry, the investigators analyzed outcomes among 1,538 M-TEER patients with VFMR and LVEF of ≤40%, including 103 subjects with LVEF of <20%, the largest cohort thus far. Not surprisingly, the very severe LVD group exhibited a higher-risk profile, manifested by more advanced ventricular remodeling and right sided dysfunction, higher natriuretic peptide concentrations, more frequent previous HF hospitalizations, and greater reliance on inotropic support. Notwithstanding, technical success approached 100%, procedural complications were uncommon, residual MR was optimal, and functional status improved significantly irrespective of LVEF strata. Moreover, LVEF below 20% did not possess an independent association with the risk for all-cause mortality, HF readmissions, or the composite of the two at 1-year. It did correlate, however, with increased rate and risk of 1-year cardiovascular mortality.

Several implications merit emphasis based on the OCEAN subanalysis. The first is that, among patients with VFMR, M-TEER feasibility and safety are not dictated by LV systolic function alone nor by device generation. Secondly, MR reduction can provide meaningful clinical benefit irrespective of LVD extent. And lastly, the latter does not obliviate the continued need for concomitant, nonvalvular oriented HF therapies, all of which should be directed at preventing cardiovascular mortality.

The OCEAN study enjoys a large sample size and prospective data collection. Also, it incorporates an internal comparator cohort with moderately reduced LV function, thus allowing for a more meaningful assessment of whether very severe LVD independently influences outcomes after M-TEER for VFMR. At the same time, some limitations should be acknowledged. As with all real-world registries, case suitability was evaluated by local Heart Teams, potentially introducing selection bias. Moreover, patients referred directly for LV assist device implantation, heart transplantation, or palliative care were excluded, and only MitraClip devices (Abbott Vascular Inc) were utilized, thereby limiting generalizability to the sickest HF population as well as to other delivery systems, respectively. Still further, echocardiographic measurements were obtained at participating centers without central core laboratory adjudication. And, most importantly, the absence of a medically treated control group precludes conclusions regarding the incremental benefit of M-TEER over contemporary medical therapy alone in this subset of patients.

The study by Omote et al^10^ represents an important step toward addressing one of the remaining uncertainties in interventional cardiology. By demonstrating high procedural safety and success, sustained symptomatic improvement, and comparable adjusted risks of death and/or HF hospitalization despite profoundly impaired ventricular function, the study reinforces the concept that very severe LVD should not, by itself, preclude consideration of TEER for VFMR. At the same time, the persistently higher cardiovascular mortality observed in the setting of very severe LVD reminds us that correcting MR does not eliminate the underlying burden of advanced myocardial disease. M-TEER should therefore be considered within the broader context of comprehensive HF management, integrated with optimized medical therapy and, when appropriate, advanced HF interventions.

As the field continues to evolve, the focus should shift from identifying arbitrary ejection fraction thresholds toward defining the clinical characteristics that identify patients most likely to benefit from intervention. A dedicated randomized trial comparing M-TEER and pharmacologic therapy with contemporary HF therapy alone in patients with LVEF below 20% represents the logical next step and will ultimately determine the role of M-TEER in this challenging and increasingly encountered patient population.

## Funding support and author disclosures

The authors have reported that they have no relationships relevant to the contents of this paper to disclose.
